# Multivariate Count Data Models for Time Series Forecasting

**DOI:** 10.3390/e23060718

**Published:** 2021-06-05

**Authors:** Yuliya Shapovalova, Nalan Baştürk, Michael Eichler

**Affiliations:** 1 Institute for Computing and Information Sciences, Radboud University Nijmegen, Toernooiveld 212, 6525 EC Nijmegen, The Netherlands; 2 School of Business and Economics, Maastricht University, Tongersestraat 53, 6211 LM Maastricht, The Netherlands; n.basturk@maastrichtuniversity.nl (N.B.); m.eichler@maastrichtuniversity.nl (M.E.)

**Keywords:** multivariate count data, INGACRCH, state-space model, bank failures, transactions

## Abstract

Count data appears in many research fields and exhibits certain features that make modeling difficult. Most popular approaches to modeling count data can be classified into observation and parameter-driven models. In this paper, we review two models from these classes: the log-linear multivariate conditional intensity model (also referred to as an integer-valued generalized autoregressive conditional heteroskedastic model) and the non-linear state-space model for count data. We compare these models in terms of forecasting performance on simulated data and two real datasets. In simulations, we consider the case of model misspecification. We find that both models have advantages in different situations, and we discuss the pros and cons of inference for both models in detail.

## 1. Introduction

Modeling time series of counts is relevant in a range of application areas, including the dynamics of the number of infectious diseases, number of road accidents or number of bank failures. In many applications, such count data dynamics are correlated across several data series. Examples include from correlated number of bank failures [1], number of crimes [2] to COVID-19 contagion dynamics [3]. The analysis of such correlations provides detailed information about the overall connectedness of the series, as well as the dynamics of an individual series conditional on the others. Several multivariate count data models have been proposed to capture the overall connectedness of multivariate count data. Each one of these models has different underlying assumptions as well as computational challenges. We present a comparative study of two families of multivariate count data models, namely State Space Models (SSM) and log-linear multivariate autoregressive conditional intensity (MACI) models, based on simulation studies and two empirical applications.

**Figure 1:**
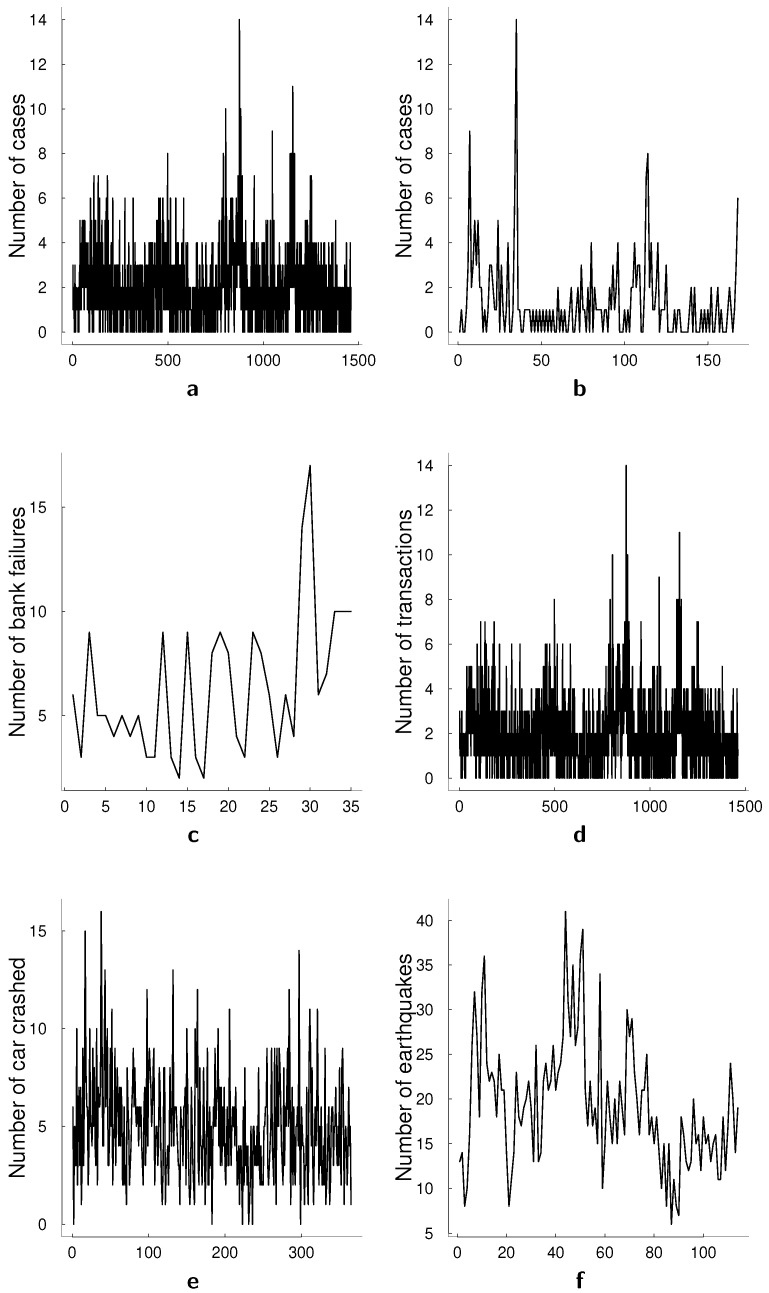
Typical examples of count data coming from applications in different scientific fields. (**a**) Monthly number of cases of poliomyelitis in the U.S. (1970–1983). (**b**) Asthma presentations at a Sydney hospital (**c**) Number of bank failures in US (**d**) Number of transactions for BMW on 30 s interval (**e**) Number of car crashes (**f**) Number of earthquakes.

We provide some examples of the count data and discuss particular properties that one desires to model when dealing with such data. In this paper, we assume that the counts are unbounded and we assume both models to be stationary. For discussion on the difference between bounded and unbounded count data and the difference of the modeling approaches for these data we refer to [4]. The top panels in Figure 1 present two conventional data sets that have been used for univariate illustrations, namely the monthly number of cases of poliomyelitis in the U.S. between 1970 and 1983, and asthma presentations at a Sydney hospital. The middle panel in Figure 1 presents the number of bank failures in the U.S. over time, a dataset that we also analyze in this paper, and the number of transactions for BMW in a 30 s interval. The bottom panel in Figure 1 presents a number of car crashes and a number of earthquakes. The former, number of car crashes over time is analyzed in Park and Lord [5] with a multivariate Poisson log-normal model with correlations for modeling the crash frequency by severity. The authors demonstrate that, accounting for the correlations in the multivariate model can improve the accuracy of the estimation. A common feature in all presented datasets is the autocorrelation present in count data over time that is visible in the time series plots. In multivariate count time series data, this correlation generalizes to a correlation between past and current values of a specific series as well as between different series.

Models for multivariate count time series typically rely on multivariate Poisson distributions, where time-variation is defined through one or more rate parameters [6]. In some cases, Gaussian approximations are used but, as has been shown in [7], this can lead to reduced performance in the risk forecasting assessment. In general, the quality of such approximations depends on a particular problem [8]. Estimation of these models is computationally demanding for high numbers of counts as the estimation relies on the sum over all counts. In addition, these models typically have positivity restrictions on the conditional intensity function that governs the Poisson process over time and the correlation between different time series. A few exceptions to the positive correlation assumption exist, see for example, [9,10].

An alternative model for the joint distribution of count data is the copula model. A number of papers proposed different copula models for multivariate count time series, see, for example, [11,12,13,14]. Copulas are generally used for modeling dependency in multiple series, which makes them attractive methods also for multiple count time series. However, several issues arise in their applications to count data such as unidentifiability and not accounting for potential overdispersion—a property that is common for count data. Genest and Nešlehová [15] provides a detailed overview of copula models for count data, and proposes and compares Bayesian and classical inferential approaches for multivariate Poisson regression. They show that computationally Bayesian and classical approaches are of a similar order.

Both approaches of modeling joint distribution of count data—multivariate Poisson distribution and copulas—can be incorporated in the autoregressive conditional intensity (ACI) framework, often also referred to as an integer-valued generalized autoregressive conditional heteroskedasticity model (INGARCH). This model belongs to the class of observation driven models as opposed to parameter driven models, a classification proposed by Cox et al. [16]. ACI models have been dominating the literature for quite a long time despite their restrictiveness: these models only allow only for positive coefficients in the equation for conditional intensity. These bounded coefficients lead to several problems besides potentially unrealistic dependence structure for some data. In particular, the problem of calculating confidence sets for the parameters that are close to or on the boundary rises and has not been yet solved in the literature. Another observation driven model that has been proposed as an alternative to ACI framework is log-linear model, see Fokianos and Tjøstheim [17], a multivariate extension of which has been considered in Doukhan et al. [10]. Even though the problem of modeling joint distribution remains, the advantage of this approach is that no restrictions on the parameter space are required due to the log-transform of the data.

Another class of models that can be considered for modeling count data, but is rarely used in the literature, is parameter driven models and, in particular, non-linear state-space models. In this framework, the observations are driven by an independent unobserved stochastic process which, for instance, can be a (vector) autoregressive process (VAR(p)). These models have been discussed extensively in the univariate case, see, for example Davis et al. [18]. However, these models are rarely used in multivariate applications due to the computationally demanding estimation methods that have to be used. To our knowledge, only one very recent study has considered them in a multivariate application Zhang et al. [19]. Non-linear state-space models are capable of modeling and inferring complex dependence structures in the data. They allow for both negative and positive contemporaneous correlation, as well as for both negative and positive Granger-causal feedback. Thereby, these models avoid the problem of modeling the joint distribution of time series of counts and provide a coherent inferential tool in the Bayesian framework. This is what distinguishes our approach from the approach discussed in Zhang et al. [19] who consider frequentist estimation of these models. We also compare SSM to log-linear models instead of MACI models since they allow for negative dependence between the intensities and hence appear to be more natural competitors of SSM models.

In this paper, we compare two classes of models, observation driven and parameter driven models, in terms of their forecasting performances. We estimate the observation driven models the quasi-maximum likelihood method. Parameter driven models, however, fit very well into the Bayesian paradigm and that is what we use for estimation. Certain advantages come together with this framework, such as those naturally obtained from the posterior distribution uncertainty about the parameters of the model and forecast of the multivariate time series [20]. We in particular use particle Markov chain Monte Carlo (pMCMC) [21] for the estimation of the parameter driven model. As is discussed in [22], pMCMC outperforms other methods (variational Bayes [23], integrated nested Laplace approximation [24] and Riemann manifold Hamiltonian Monte Carlo [25]) in terms of parameter estimation. There are other recent methods for the estimation of the state-space models such as auxiliary likelihood-based approximate Bayesian computation [26] and variational Sequential Monte Carlo [27], but their performance has to be investigated further, which is outside of the scope of this paper.

We present a set of simulation studies to show how these models perform when they are correctly specified and misspecified. The simulation results show that, as expected, the correctly specified models perform generally well, but there are exceptions. Particularly, parameter driven models have better forecast performances in some simulations even if they are misspecified. In addition to these simulation studies, we compare the performances of these models in two real data applications. The two data sets we analyze exhibit different sample sizes, standard deviation, dispersion and maximum counts. We show that the overall forecast performances of the models can be very different, depending on the applications. Furthermore, for the second data set we analyze, we find that observation driven models capture extreme data values better than parameter driven models.

The remainder of this paper is as follows: Section 2 and Section 3 summarize observation and parameter driven models, respectively. Section 4 presents the model and forecast comparison tools we use for multivariate count data models. Section 5 presents simulation results. Section 6 presents results from applications to two data sets. Finally, Section 7 concludes the paper.

## 2. Observation Driven Models

In this section, we summarize two observation driven models: multivariate autoregressive conditional intensity model and log-linear analog of it. Both of these models are characterized by the dynamics that depend on the past of the process itself and some noise. Both models have been considered in Doukhan et al. [10], where the authors discussed some theoretical properties and proposed to use copula approach for modeling joint count distribution. Copulas are flexible tools for modeling dependence structure but their use in count time series models brings challenges. We first summarize the use of Poisson distribution for count data, analyze both models under an independence assumption in the Poisson random variables, and at the end of this section, we discuss the extension of modeling multiple count time series with multivariate Poisson distribution.

### 2.1. Poisson Distribution

Many of the count time series models take their origins in the idea of Poisson regression model, an extensive overview of these models is given in Fokianos [28]. Specifically, both models considered in this section as well as the parameter driven models in Section 3 rely on Poisson distributions. We therefore first provide some background on the Poisson distribution. Poisson distribution has played an important role in modeling count time series data as its interpretation is the number of independent events that occur in a time period. The Poisson distribution is defined for a random variable *x* takes integer values in {0,1,…}. The mean of the distribution, λ, describes the average occurrences per interval, the distribution has the equi-dispersion property since the variance its variance is also λ, and the probability mass function (pmf) of the distribution is

(1) $$p\left(x\right)=\frac{\lambda^{x}e^{-\lambda }}{x!},\;\;\;x=0,1,2,3\ldots ,$$

with E(x)=Var(x)=λ.

For the simple multivariate case, two Poisson random variables, say $x_{1}$ and $x_{2}$, the joint pmf reads

(2) $$p\left(x_{1},x_{2}\right)=\prod_{i=1}^{2}\frac{e^{-\lambda_{i}}\lambda_{i}^{x_{i}}}{x_{i}!}.$$

The multivariate extension in (2) is rather naive due to the underlying independence assumption between $x_{1}$ and $x_{2}$. Such a model would ignore potential dependency of the real world data, thus is potentially not suitable for the majority of applications. One way to use the Poisson distribution for modeling count data in multivariate case and incorporate correlation structure is through the so-called *trivariate reduction* [13,29]. The idea is that correlation can be modeled through the third Poisson variable. Assume we have three independent random variables $x_{i}∼\mathrm{Poisson}\left(\lambda_{t,i}-\varphi \right)$, where $0\leq \varphi \leq \min\{\lambda_{t,1},\lambda_{t,2}\}$. Define $Y_{t,1}=X_{1}+X_{2}$ and $Y_{t,2}=X_{2}+X_{3}$. In this way, the random variable $X_{2}$ is exploited to model the dependence between $Y_{1}$ and $Y_{2}$. The restriction of this approach is that the correlation is the same between all the series (in case one wants to model the systems beyond bivariate case) and the dependence can only be positive. We further discuss the trivariate reduction technique in the context of ACI/INGARCH models. In particular, the difficulties of extending this to higher dimensions is of interest and presents one with a challenging task.

### 2.2. MACI (INGARCH)

The Poisson integer-valued generalized autoregressive conditional heteroscedastic process (INGARCH) models [30]—also called multivariate autoregressive conditional intensity models (MACI) in the literature—are built upon GARCH framework and are capable of capturing time series properties of count data. As for GARCH-type models, it is assumed that the conditional mean of the process at time *t* depends on the value of the process at period t−1 and its conditional mean at time t−1. The time series of counts follow Poisson process with the conditional mean $\lambda_{t}$, that is,

(3) $$X_{i,t}|F_{t-1}∼\mathrm{Poisson}\left(\lambda_{i,t}\right),\;\;\;i=1,\ldots ,n.$$

The corresponding joint pmf reads

(4) $$P\left(X_{1t}=x_{1t},\ldots ,X_{nt}=x_{nt}|F_{t-1}\right)=\prod_{i=1}^{n}\frac{e^{-\lambda_{it}}\lambda_{it}^{x_{it}}}{x_{it}!}.$$

The dynamics of the conditional intensity $\lambda_{t}=E\left[X_{t}|F_{t-1}\right]$ follows

(5) $$\lambda_{t}=\omega +\sum_{i=1}^{n}A^{i}\lambda_{t-i}+\sum_{j=1}^{q}B^{j}X_{t-j}.$$

Note that the elements of ω, $a^{i}$, $b^{j}$ are assumed to be positive to ensure the positivity of the intensity process λ. (Doukhan et al. [10] argue that the condition $||A+B|{|}_{2}<1$ guarantees stationarity.) In addition, we assume no contemporaneous correlation in the counts. Consider the bivariate case for the conditional intensity process

(6) $$\left[\begin{array}{c} \lambda_{1t}\\ \lambda_{2t} \end{array}\right]=\left[\begin{array}{c} \omega_{1}\\ \omega_{2} \end{array}\right]+\left[\begin{array}{cc} a_{11} & a_{12}\\ a_{21} & a_{22} \end{array}\right]\left[\begin{array}{c} \lambda_{1t-i}\\ \lambda_{2t-i} \end{array}\right]+\left[\begin{array}{cc} b_{11} & b_{12}\\ b_{21} & b_{22} \end{array}\right]\left[\begin{array}{c} X_{1t-j}\\ X_{2t-j} \end{array}\right],\;t=0,\pm 1,\pm 2,\ldots .$$

From Equation (6) it is clear that when A and B are diagonal, there is no dependence structure between the intensities. Further, when $a_{12}=0$ and $b_{12}=0$ then the intensity of the first process, $\lambda_{1,t}$, depends only on its own past while the second process can depend on the dynamics of the first one. Finally, if we restrict A to be diagonal and B to be non-diagonal, every intensity process would depend on its past and possibly on the past of all of the observations. This constraint is relevant when one wants to apply graphical modeling to this problem.

### 2.3. Quasi-Maximum Likelihood for MACI Models

In this section, we discuss how the inference for MACI/INGARCH models can be executed. The details for the multivariate case have also been discussed in Doukhan et al. [10]. For these models, we make use of the classical estimation framework and in particular use quasi-maximum likelihood estimation. The conditional quasi-likelihood for this model and θ reads

(7) $$L\left(\theta \right)=\prod_{t=1}^{T}\prod_{i=1}^{n}\left(\frac{\exp\left(-\lambda_{i,t}\left(\theta \right)\right)\lambda_{i,t}^{x_{i,t}}\left(\theta \right)}{x_{i,t}!}\right),$$

where θ are the parameters of interest. It follows the the quasi log-likelihood function is

(8) $$l\left(\theta \right)=\sum_{t=1}^{T}\sum_{i=1}^{n}\left(x_{i,t}\log\lambda_{i,t}\left(\theta \right)-\lambda_{i,t}\left(\theta \right)\right),$$

and the corresponding score function reads

(9) $$\begin{array}{cc} S_{T}\left(\theta \right)= & \sum_{t=1}^{T}\sum_{i=1}^{n}\left(\frac{x_{i,t}}{\lambda_{i,t}}-1\right)\frac{\partial \lambda_{i,t}\left(\theta \right)}{\partial \theta }\\ = & \sum_{t=1}^{T}\frac{\partial \lambda_{t}^{T}\left(\theta \right)}{\partial \theta }D_{t}^{-1}\left(\theta \right)\left(X_{t}-\lambda_{t}\left(\theta \right)\right)\equiv \sum_{t=1}^{T}s_{t}\left(\theta \right), \end{array}$$

where $\partial \lambda_{t}/\partial \theta^{T}$ is n×d matrix with d≡n(1+2n) being the dimension of the parameter vector θ, $D_{t}$ is an n×n diagonal matrix and its diagonal elements are $\lambda_{i,t}\left(\theta \right)$, i=1,2,…,n, and $X_{t}$ consists of elements $x_{i,t}$, i=1,2,…,n, t=1,2,…,T. Thus the recursions for the quasi-maximum likelihood estimation follow

(10) $$\frac{\partial \lambda_{t}}{\partial \omega^{T}}=I_{n}+A\frac{\partial \lambda_{t-1}}{\partial \omega^{T}},$$

(11) $$\frac{\partial \lambda_{t}}{\partial vec^{T}\left(A\right)}={\left(\lambda_{t-1}⊗I_{n}\right)}^{T}+A\frac{\partial \lambda_{t-1}}{\partial vec^{T}\left(A\right)},$$

(12) $$\frac{\partial \lambda_{t}}{\partial vec^{T}\left(B\right)}={\left(X_{t-1}⊗I_{n}\right)}^{T}+A\frac{\partial \lambda_{t-1}}{\partial vec^{T}\left(B\right)}.$$

Finally, the Hessian matrix and the conditional information matrix are correspondingly

(13) $$\begin{array}{c} H_{T}\left(\theta \right)=\sum_{t=1}^{T}\sum_{i=1}^{n}\frac{x_{i,t}}{\lambda_{i,t}^{2}\left(\theta \right)}\frac{\partial \lambda_{i,t}\left(\theta \right)}{\partial \theta }\frac{\partial \lambda_{i,t}\left(\theta \right)}{\partial \theta^{T}}-\sum_{t=1}^{T}\sum_{i=1}^{n}\left(\frac{x_{i,t}}{\lambda_{i,t}\left(\theta \right)}-1\right)\frac{\partial^{2}\lambda_{i,t}\left(\theta \right)}{\partial \theta \partial \theta^{T}}, \end{array}$$

(14) $$G_{T}=\sum_{t=1}^{T}\frac{\partial \lambda_{t}^{T}\left(\theta \right)}{\partial \theta }D_{t}^{-1}\left(\theta \right)\Sigma_{t}D_{t}^{-1}\left(\theta \right)\frac{\lambda_{t}\left(\theta \right)}{\partial \theta^{T}}.$$

Further, one can show that $S_{n}\left(\theta \right)=0$ has a unique solution, $\hat{\theta }$, which is strongly consistent and asymptotically normal. For further details of these properties, we refer the reader to Doukhan et al. [10]. However, that theoretical properties of $\hat{\theta }$ are proven under assumption that the true value $\theta_{0}$ belongs to the interior of the parameter space Θ. The problems certainly arise when the true parameter is close or on the boundary of the parameter space. Dealing with the theoretical problems of the constrained optimization and parameters near or on the boundary of parameter space is out of the scope of this paper and generally establishing the theory for this case is a complicated task. One of the possible solutions is to exploit bootstrap methods for this task, see Hilmer et al. [31] for a comparison of some bootstrap methods related to this sort of problem and review of other possible approaches.

### 2.4. Log-Linear Autoregressive Model

Log-linear models have appeared in the count data literature in the recent years [10] and have good potential since they allow for both positive/negative correlation and avoid parameter boundary problems which MACI models suffer from.

(15) $$X_{i,t}|F_{t-1}^{X,\lambda }∼\mathrm{Poisson}\left(\lambda_{i,t}\right),$$

(16) $$\nu_{t}=\omega +A\nu_{t-1}+B\log\left(X_{t-1}+1_{n}\right),\;\;\;t\geq 1,$$

where $F_{t-1}^{Y,\lambda }$ is the σ−field generated by $\left\{X_{0},\ldots ,X_{t},\lambda_{0}\right\}$, $1_{n}$ is the *n*-dimensional vector of ones, $\nu_{t}\equiv \log\lambda_{t}$. Parameters of this model, ω, A, and B, do not have to be positive which makes this model more attractive than MACI.

### 2.5. Quasi-Maximum Likelihood for Log-Linear Models

The inference in log-linear models is very similar to the quasi-maximum likelihood approach derived for MACI models in Section 2.3. Only minor adjustments have to be made in corresponding recursions [10]. In particular, the score function for the log-linear model reads

(17) $$S_{T}\left(\theta \right)=\sum_{t=1}^{T}\sum_{i=1}^{n}\left(x_{i,t}-\exp\left(\nu_{i,t}\left(\theta \right)\right)\right)\frac{\partial \nu_{i,t}\left(\theta \right)}{\partial \theta }=\sum_{t=1}^{T}\frac{\partial \nu_{t}^{T}\left(\theta \right)}{\partial \theta }\left(X_{t}-\exp\left(\nu_{t}\left(\theta \right)\right)\right),$$

the Hessian matrix is

(18) $$H_{T}\left(\theta \right)=\sum_{t=1}^{T}\sum_{i=1}^{n}\exp\left(\nu_{i,t}\left(\theta \right)\right)\frac{\partial \nu_{i,t}\left(\theta \right)}{\partial \theta }\frac{\partial \nu_{i,t}\left(\theta \right)}{\partial \theta^{T}}-\sum_{t=1}^{T}\sum_{i=1}^{n}\left(x_{i,t}-\exp\left(\nu_{i,t}\left(\theta \right)\right)\right)\frac{\partial^{2}\nu_{i,t}\left(\theta \right)}{\partial \theta \partial \theta^{T}},$$

and the conditional information matrix for the log-linear model reads

(19) $$G_{T}\left(\theta \right)=\sum_{t=1}^{T}\sum_{i=1}^{n}\exp\left(\nu_{i,t}\left(\theta \right)\right)\frac{\partial \nu_{i,t}\left(\theta \right)}{\partial \theta }\frac{\partial \nu_{i,t}\left(\theta \right)}{\partial \theta^{T}}.$$

Doukhan et al. [10] prove theoretical properties of this model. In particular, they show that there exists a unique solution $\hat{\theta }$ which is strongly consistent and asymptotically normal. The authors also show that the condition $\sum_{j=0}^{\infty }||A^{j}B|{|}_{2}<1$ guarantees both stationarity and weak dependence.

### 2.6. Multivariate Poisson Distribution

To allow for contemporaneous correlation, we need to use trivariate reduction technique discussed before. We consider the bivariate case to give an example, assume that there are three independent random variables $Y_{1}$, $Y_{2}$, $Y_{3}$ with positive means $\lambda_{1}$, $\lambda_{2}$, $\lambda_{3}$ respectively. Define random variables $X_{1}=Y_{1}+Y_{3}$ and $X_{2}=Y_{2}+Y_{3}$. The new random variables will have means $\lambda_{1}+\lambda_{3}$ and $\lambda_{2}+\lambda_{3}$, where $\lambda_{3}$ would also correspond to the covariance between $X_{1}$ and $X_{2}$. The covariance is clearly restricted to be positive, while correlation will lie between 0 and $\min\{\frac{\sqrt{\lambda_{1}+\lambda_{3}}}{\sqrt{\lambda_{2}+\lambda_{3}}},\frac{\sqrt{\lambda_{2}+\lambda_{3}}}{\sqrt{\lambda_{1}+\lambda_{3}}}\}$. Thereby the joint pmf of interest, alternative to what we have in Equation (3), becomes

(20) $$\begin{array}{cc} P(X_{1t}=x_{1t},X_{2t}=x_{2t}|F_{t-1})= & e^{-(\lambda_{1}+\lambda_{2}+\lambda_{3})}\frac{\lambda_{1}^{x_{1}}\lambda_{2}^{x_{2}}}{x_{1}!x_{2}!}\\ \; & \times \sum_{i=0}^{\min(x_{1},x_{2})}\left(\begin{array}{c} x_{1}\\ i \end{array}\right)\left(\begin{array}{c} x_{2}\\ i \end{array}\right)i!{\left(\frac{\lambda_{3}}{\lambda_{1}\lambda_{2}}\right)}^{i}. \end{array}$$

Extending this approach to contemporaneous correlation in higher dimensions is not trivial. Suppose that we would like to model *n* Poisson random variables, thus $X_{i}∼\mathrm{Poisson}\left(\lambda_{i}\right)$, i=1,…,n. By defining a random variable $X_{0}∼\mathrm{Poisson}\left(\lambda_{0}\right)$ and extending the argument of the trivariate reduction we can define random variables $X_{1}=Y_{1}+Y_{0},X_{2}=Y_{2}+Y_{0},\ldots ,X_{n}=Y_{n}+Y_{0}$. The joint pmf is

(21) $$\begin{array}{cc} P(X_{1}=x_{1},X_{2}=x_{2},\ldots ,X_{n}=x_{n})= & \exp\left(-\sum_{i=1}^{n}\lambda_{i}\right)\prod_{i=1}^{n}\frac{\lambda_{i}^{x_{i}}}{x_{i}!}\\ \; & \times \sum_{i=0}^{m}\sum_{j=1}^{n}\left(\begin{array}{c} x_{j}\\ i \end{array}\right)i!{\left(\frac{\lambda_{0}}{\prod_{i=1}^{n}\lambda_{i}}\right)}^{i}, \end{array}$$

where $m=\min(x_{1},x_{2},\ldots ,x_{n})$. This approach assumes that the covariance is the same for all the pairs of Poisson random variables which is very restrictive. Karlis and Meligkotsidou [9] consider the case with richer covariance structure which we discuss further. For simplicity, assume we want to model trivariate time series of counts $Y_{1},Y_{2},Y_{3}$. As before, let us specify through $X_{i}$ and $X_{ij}$ univariate Poisson random variables, i.e., $X_{i}∼\mathrm{Poisson}\left(\lambda_{i}\right)$ and $X_{ij}∼\mathrm{Poisson}\left(\lambda_{ij}\right)$ with i,j∈{1,2,3}, i<j. Then the random variables $Y_{i}$ with i∈{1,2,3} are defined in the following way

(22) $$\begin{array}{c} Y_{1}=X_{1}+X_{12}+X_{13},\\ Y_{2}=X_{2}+X_{12}+X_{23},\\ Y_{3}=X_{3}+X_{13}+X_{23}. \end{array}$$

Thus, $Y_{i}∼\mathrm{Poisson}\left(\lambda_{i}+\lambda_{ij}+\lambda_{ik}\right)$, where i,j,k∈{1,2,3}, i≠j≠k. Finally, these random variables follow the Poisson distribution with $\lambda =(\lambda_{1},\lambda_{2},\lambda_{3},\lambda_{12},\lambda_{13},\lambda_{23})$, and hence with the mean vector $A\lambda ={\left(\lambda_{1}+\lambda_{12}+\lambda_{13},\lambda_{2}+\lambda_{12}+\lambda_{23},\lambda_{3}+\lambda_{13}+\lambda_{23}\right)}^{\prime }$. The variance-covariance matrix for this distribution is given by

(23) $$A\Sigma A^{\prime }=\left(\begin{array}{ccc} \lambda_{1}+\lambda_{12}+\lambda_{13} & \lambda_{12} & \lambda_{13}\\ \lambda_{12} & \lambda_{2}+\lambda_{12}+\lambda_{23} & \lambda_{23}\\ \lambda_{13} & \lambda_{23} & \lambda_{3}+\lambda_{13}+\lambda_{23} \end{array}\right)$$

It is clear from the above examples that the modeling of the time series of counts with multivariate Poisson distribution in higher dimensions is restrictive and cumbersome. It is restrictive, since it allows only for positive dependency in the data, which can be unreasonable for real-world applications. It is cumbersome since the method is only computationally tractable when one has low counts data, see Equation (21) in which the number of terms in the sum depends on the number of observed counts. Methods such as expectation maximization can be applied in this case but they are not trivial and stable in case of high counts. Moreover, in this case, incorporation of the multivariate Poisson distribution into MACI or log-linear models also affects computational speed substantially, and these models lose their attractiveness over more complex models such as nonlinear state-space models in the next section.

## 3. Parameter Driven Model: Nonlinear State-Space Model

The advantage of parameter driven models is the clear interpretability of the model parameters and the high degree of flexibility. The model can easily incorporate different distributions and extends easily to the multivariate framework. Moreover, in the Bayesian framework, we have coherent inferential tools derived from the posterior distributions of the parameters, such as highest posterior density intervals. These models also take into account uncertainty about the parameters which is incorporated into predictions. The disadvantage of this approach is challenging estimation procedures that are computationally intensive. Hence, even though theoretically estimation methodologies are possible to extend to any dimension, in practice that is not feasible due to the time constraints. In this paper, we estimate the parameter-driven model for multivariate count data using Sequential Monte Carlo and particle Markov Chain Monte Carlo. These methods became popular with the availability of more computational power. They are restricted in some ways, and we will discuss these restrictions in the next subsections after introducing the nonlinear state space model (SSM), which we will compare to the observation driven models.

### 3.1. Multivariate SSM

A state-space model is usually presented by an observation equation and a state equation. The state equation represents a latent process, say $h_{t}$, which drives the dynamics of observations $y_{t}$. In the multivariate SSM for count data below, this dependence between the observations and the state is nonlinear

(24) $$X_{it}∼\mathrm{Poisson}\left(\lambda_{it}\right),\;\;\;i=1,2,\ldots ,n$$

(25) $$\lambda_{t}=\beta \exp\left(h_{t}\right)$$

(26) $$h_{t}=\sum_{i=1}^{n}\Phi^{i}h_{t-i}+\eta_{t},\;\Sigma_{\eta }=\left(\begin{array}{cccc} \sigma_{\eta_{1}}^{2} & \rho_{\eta_{12}} & \ldots & \rho_{\eta_{1n}}\\ \rho_{\eta_{21}} & \sigma_{\eta_{2}}^{2} & \ldots & \rho_{\eta_{2n}}\\ \vdots & \vdots & \ddots & \vdots \\ \rho_{\eta_{n1}} & \ldots & \ldots & \sigma_{\eta_{n}}^{2}, \end{array}\right)$$

where $\eta_{t}∼N\left(0,\Sigma_{\eta }\right)$. Equation (24) shows that the observations have Poisson distribution with mean $\lambda_{t}$ defined through the Equation (25), and $\lambda_{t}$ nonlinearly depends on the latent process $h_{t}$ which is defined through Equation (26). Note that the latent process is defined through a VAR(p) process, and hence corresponding theory applies. In particular, the stationarity condition is that the roots of the Equation (27) must lie outside the unit circle

(27) $$|\lambda^{p}I_{n}-\lambda^{p-1}\Phi_{1}-\ldots \Phi_{p}|=0.$$

The dependence structure between counts is modeled through the dependence in the latent process. Conditioned on the latent process ${\left\{h_{t}\right\}}_{t=1}^{T}$ the observations ${\left\{X_{t}\right\}}_{t=1}^{T}$ are independent. Furthermore, since the latent process of the model is a VAR(p), we can account for various dependence structures: positive and negative contemporaneous correlation, and positive and negative Granger-causal feedback.

These models are challenging to estimate, and an assumption of p=1 can simplify the inference. (For extending the model to p>1 we advise the reader to consider using sparse priors, such as Minnesota prior, spike and slab or horseshoe prior.) Bivariate specification of the nonlinear state-space model with the lag p=1 reads

(28) $$X_{it}∼\mathrm{Poisson}\left(\lambda_{it}\right),\;\;\;i=1,2$$

(29) $$\lambda_{it}=\beta_{i}\exp\left(h_{it}\right),\;\;\;i=1,2.$$

(30) $$\left(\begin{array}{c} h_{1,t+1}\\ h_{2,t+1} \end{array}\right)=\left(\begin{array}{cc} \phi_{11} & \phi_{12}\\ \phi_{21} & \phi_{22} \end{array}\right)\left(\begin{array}{c} h_{1,t}\\ h_{2,t} \end{array}\right)+\left(\begin{array}{c} \eta_{1t+1}\\ \eta_{2t+1} \end{array}\right),\;\Sigma_{\eta }=\left(\begin{array}{cc} \sigma_{\eta_{1}}^{2} & \rho_{\eta_{12}}\\ \rho_{\eta_{21}} & \sigma_{\eta_{2}}^{2}. \end{array}\right)$$

The dependence structure between series is described by the Granger-causal relationship in the latent processes $h_{it}$ and contemporaneous relations that are incorporated in $\Sigma_{\eta }$. For example, we say that $h_{2,t}$ does not Granger-cause $h_{1,t}$ if $\phi_{12}=0$. Correlation coefficient ρ in this model allows us to model both positive and negative correlation between the counts.

### 3.2. Bayesian Inference in Multivariate SSM

The estimation of nonlinear state-space models naturally fits into the Bayesian framework. The presence of the unobservable process in the model and nonlinear dependence of the observations on this unobservable process leads to an intractable likelihood and posterior. For this reason, and due to the nonlinear SSM structure, we use particle Markov Chain Monte Carlo (PMCMC) for the estimation of the posterior distribution of the model parameters Andrieu et al. [21]. The method consists of two parts. First, the likelihood is estimated in a sequential manner through a *particle filter*. Second, this estimate is used within an MCMC sampler, in our case Metropolis-Hastings algorithm. An extensive introduction to nonlinear state-space models and particle filtering can be found, for example, in Särkkä [32].

Recall the Bayes rule on which the inference is based

(31) $$p\left(h_{1:T}|x_{1:T}\right)=\frac{p\left(x_{1:T}|h_{1:T}\right)\pi \left(h_{1:T}\right)}{p(x_{1:T})},$$

where $\pi (h_{1:T})$ is the prior distribution on the parameters of the volatility process defined by the dynamic model, $p(x_{1:T}|h_{1:T})$ is the likelihood of the observations, $p(x_{1:T})$ is the normalization constant that is ignored during the inference. Thus, we use Bayes rule in proportionality terms

(32) $$p\left(h_{1:T}|x_{1:T}\right)\propto p\left(x_{1:T}|h_{1:T}\right)\pi \left(h_{1:T}\right).$$

We use particle Metropolis-Hastings to estimate the posterior distribution of the parameters of the model since neither the likelihood nor the posterior are available in closed form. We use Metropolis-Hastings algorithm to sample from the posterior of the parameters. Algorithm 1 presents an iteration of the Metropolis-Hastings algorithm. At every iteration of the algorithm we make a new proposal $\theta_{i}^{c}$ for the parameter vector using a proposal mechanism $q(\cdot |\theta^{\left(i\right)})$. Then we accept the proposed candidate $\theta_{i}^{c}$ with probability α. The acceptance probability in Algorithm 1 depends on $p(\theta ,h_{1:T}|x_{1:T})$—target distribution—and q(·)—proposal distribution. How well we manage to explore the posterior distribution depends on the acceptance rate of the algorithm. When the acceptance rate is too high it is often related to too small proposal steps, and the other way around. Overall, either case slows down the convergence of the Markov Chain. General advice for the optimal performance of the algorithm is an acceptance rate that is around 0.234 [33].
**Algorithm 1** Particle Metropolis-Hastings Algorithm1: Given $\theta^{\left(i\right)}$,2: Generate $\theta_{i}^{c}∼q\left(\cdot |\theta^{\left(i\right)}\right)$,3: Take$$\;\theta^{\left(i+1\right)}=\left\{\begin{array}{c} \theta_{i}^{c},\;\;\;\mathrm{with}\;\mathrm{probability}\;\;\;\rho \left(\theta^{\left(i\right)},\theta_{i}^{c}\right)\\ \theta^{\left(i\right)}\;\;\;\mathrm{with}\;\mathrm{probability}\;\;\;1-\rho \left(\theta^{\left(i\right)},\theta_{i}^{c}\right), \end{array}\right.$$  where$$\;\rho \left(\theta^{\left(i\right)},\theta_{t}^{c}\right)=\min\left(\frac{p_{\theta_{i}}^{c}\left(x_{1:T}\right)}{p_{\theta }^{\left(i\right)}\left(x_{1:T}\right)}\frac{\pi (\theta_{i}^{c})}{\pi (\theta_{i})}\frac{q(\theta^{\left(i\right)}|\theta_{i}^{c})}{q(\theta_{i}^{c}|\theta^{\left(i\right)})},1\right)$$

Using Algorithm 1 we obtain samples from otherwise intractable distribution $p(\theta ,h_{1:T}|x_{1:T})$. Note, that $p_{\theta_{i}}^{c}\left(x_{1:T}\right)$ and $p_{\theta }^{\left(i\right)}\left(x_{1:T}\right)$ are also intractable. Thus, in practice we substitute them with the estimates ${\hat{p}}_{\theta_{i}^{c}}\left(x_{1:T}\right)$ and ${\hat{p}}_{\theta^{\left(i\right)}}\left(x_{1:T}\right)$ obtained with Sequential Monte Carlo.

We further discuss how $p_{\theta }\left(x_{1:T}\right)$ can be estimated.

### 3.3. Estimation of the Likelihood with SMC

Sampling from the posterior distribution with algorithms such as Metropolis-Hastings requires evaluationg the likelihood. In case of non-linear state-space models, this likelihood evaluation is not straightforward since the likelihood is a high dimensional integral

(33) $$\begin{array}{c} L\left(x_{1:T}\right)=\int p\left(x_{1:T},h_{1:T}\right)dh_{1:T}=\int p\left(x_{1:T}|h_{1:T}\right)p\left(h_{1:T}\right)dh_{1:T}\\ =\int p\left(x_{1}|h_{1}\right)p\left(h_{1}\right)\prod_{t=2}^{T}p\left(x_{t}|h_{t}\right)p\left(h_{t}|h_{t-1}\right)dh_{1}\ldots h_{T}, \end{array}$$

and this likelihood is not analytically tractable. Instead of relying on an analytical result, the integral from Equation (33) can be approximated using Sequential Monte Carlo methods, also known as particle filters. This estimate of the likelihood is then used in Algoperithm 1 as ${\hat{p}}_{\theta }\left(x_{1:T}\right)$. Algorithm 2 represents a simple version of a particle filter which we use in this paper. The algorithm consists of three main steps: prediction, updating and resampling. In the prediction step we sample *N* particles according to the assumed dynamics of the latent process $p(h_{t}|h_{t-1})$. Then we weight each particle according to the distribution of the data given the latent state $p(x_{t}|h_{t})$. Finally, in the resampling step we resample the particles according to these weights. Resampling step is meant to solve the known problem of particle degeneracy: without resampling we end up only with a few particles with high weights over time.
**Algorithm 2** Bootstrap Particle Filter with resampling1:Draw a new point $h_{t}^{\left(i\right)}$ for each point in the sample set $\left\{h_{k-1}^{\left(i\right)}:i=1,\ldots ,N\right\}$ from the dynamic model:
$h_{t}^{\left(i\right)}∼p\left(h_{t}|h_{t-1}^{\left(i\right)}\right),\;\;\;i=1,\ldots ,N.$2:Calculate the weights
$$\omega_{t}^{\left(i\right)}∼p\left(x_{t}|h_{t}^{\left(i\right)}\right),\;\;\;,1,\ldots ,N,$$
and normalize them to sum to unity.3:Compute the estimate of $p(x_{t}|x_{1:t-1},\theta )$ as
$$p\left(x_{t}|x_{1:t-1},\theta \right)=\sum_{i}\log\omega_{t}^{(i).}$$Perform resampling:4:Interpret each weight $\omega_{t}^{\left(i\right)}$ as the probability of obtaining the sample index *i* in the set $\left\{h_{t}^{\left(i\right)}:i=1,\ldots ,N\right\}$.5:Draw *N* samples from that discrete distribution defined by the weights and replace the old samples set with this new one.

The particle filter provides us with the sequence of distributions $p(h_{t}|x_{t})$, however due to particle degeneracy problem discussed previously, sampling from $p(h_{1:T}|x_{1:T})$ and approximating $p(h_{k}|x_{1:T})$, k=1,…,T, is inefficient. One of the possible solutions to this problem is using so called forward filtering—backward smoothing recursions [34]. The algorithms starts with sampling $h_{T}^{*}∼\hat{p}\left(h_{T}|x_{1:T}\right)$, and then backwards for k=T−1,T−2,…,1, we sample $h_{k}^{*}∼\hat{p}\left(h_{k}|h_{k+1}^{*},x_{1:n}\right)$. Then we can approximate the distribution $\hat{p}\left(h_{k}|x_{1:T}\right)$ as follows

(34) $$\begin{array}{cc} \hat{p}\left(h_{k}|x_{1:T}\right)= & \sum_{i=1}^{N}W_{k}^{i}\times \left[\sum_{j=1}^{N}W_{k+1|T}^{j}\frac{f(h_{k+1}^{*,j}|h_{k}^{*,i})}{\left[\sum_{l=1}^{N}W_{k}^{l}f\left(h_{k+1}^{*,j}|h_{k}^{*,l}\right)\right]}\delta_{h_{k}^{*,i}}\left(h_{k}\right)\right]\\ = & \sum_{i=1}^{N}W_{k|T}^{i}\delta_{h_{k}^{*,i}}\left(h_{k}\right). \end{array}$$

The smoothing comes at cost of O(NT) operations to sample a path from $p(h_{1:T}|x_{1:T})$ and $O(N^{2}T)$ operations to approximate $p(h_{k}|x_{1:T})$. This method works very well, in particular when dealing with large sample sizes. However, its performance comes at the price of a high computational costs. Thereby, it is generally recommended to use it when the sample size of the data is large and hence Sequential Monte Carlo is more likely to suffer from particle degeneracy. There exist other methods that are computationally less expensive [34]. However, in higher dimensions, they would be less reliable, and it would be recommendable to use more expensive methods.

### 3.4. Forecasting with SSM

One of the interests of statistical inference is the ability to perform forecasting exercises and thus handling the uncertainty about the future in the best possible way. In this section, we will discuss how forecasting task fits into the Bayesian framework, and in particular how it can be done for models of our interest.

Recall that we estimated multivariate SSM model for count data in the Bayesian framework. Observing the data $x=(x_{1},\ldots ,x_{T})$ we estimated the posterior distribution of the parameters in our model using particle Markov Chain Monte Carlo methods p(θ|x). Suppose that we are interested in predicting the next *s* observations, that is, $x_{T+1},\ldots ,x_{T+s}$. First, note that the prediction equation for the next step reads

(35) $$p\left(x_{t+1}|x_{t}\right)=\int p\left(x_{t+1}|h_{t+1}\right)p\left(h_{t+1}|x_{t}\right)dh_{t}.$$

In the framework of particle Markov Chain Monte Carlo, it is natural to adopt a sequential nature of SMC and the fact that we obtain posterior draws in the MCMC part of the algorithm. Thereby, for every vector θ of the parameters drawn in the MCMC, we can propagate the particles obtained at time *T* and based on those make one-step ahead forecast. The similar idea extends to *s*-steps ahead forecasts. In this case the uncertainty about the parameters is included in the forecasts.

When forecasting, the most natural but cumbersome approach would be to update the posterior distribution every time we get a new observation. It would mean that we generate as many MCMC chains as we have steps for forecasting. This can be carried out in a straightforward way by re-estimating the posterior distribution every time or more efficiently by incorporating this into the SMC framework. However, for large enough samples, adding an extra estimation into the PMCMC framework should not change the results substantially. Ignoring this update also makes the forecasting performance of the frequentist and Bayesian approaches more comparable. Both frameworks are estimated in different paradigms. While SSM naturally fits into the Bayesian paradigm, the log-linear model is usually estimated using frequentist methods (quasi-maximum likelihood in this case). Since our goal is not to compare the two approaches to statistics, this design of forecasting exercise is more fair.

**Figure 2:**
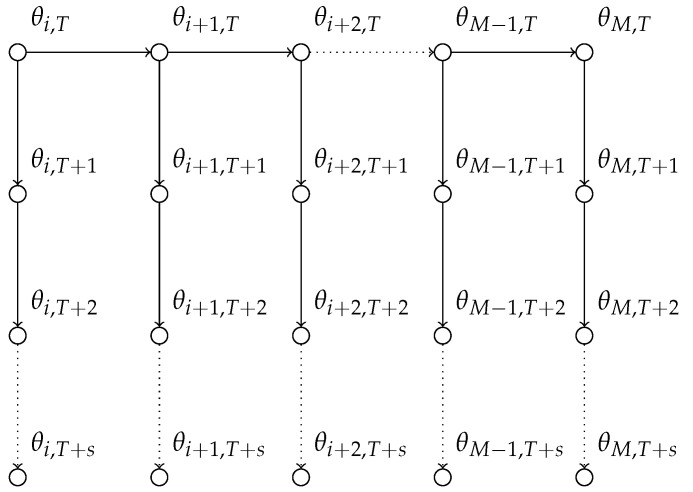
Visual representation of forecasting with the state-space model for count data.

Figure 2 illustrates the forecasting approach we undertake with the state-space model in a graphical representation. In particular, one can see that we do not re-estimate the posterior distribution every time we receive a data point.

## 4. Model Comparison and Prediction Assessment

We next summarize the measures using which we compare the models in Section 2 and Section 3. Observation driven models in this comparison are represented by the log-linear autoregressive model. The log-linear autoregressive model is more flexible than the MACI model since it can account for a negative correlation and thus it is a fairer competitor. The parameter driven approach is the state-space model, where observations are generated from the Poisson distribution and dependency is modeled through a latent process. Note that, for the latter framework, we follow a fully Bayesian approach. Thereby, we compare these two classes of models by model fit and forecasting performance criteria. The standard measures to access the model fit and forecast accuracy would be Mean Squared Error (MSE) and Mean Absolute Error (MAE) defined in Equation (36) respectively.

(36) $$MSE=\frac{1}{s}\sum_{i=1}^{s}{\left(x_{i}-\hat{x_{i}}\right)}^{2},\;MAE=\frac{1}{s}\sum_{i=1}^{s}{\left|x_{i}-\hat{x_{i}}\right|}^{2}.$$

In Czado et al. [35] the authors propose comparing forecast performance using some scoring rules. To define scoring rules, let *P* be the predictive distribution and *x* be the counts; then the penalty is defined through s(P,x). Table 1 presents some of the scoring rules one can use for comparing the performance of count data models.

Note, that in practice, one calculates the mean score

(37) $$S=\frac{1}{n}\sum_{i=1}^{n}s\left(P^{\left(i\right)},x^{\left(i\right)}\right).$$

To compare our results with the conclusions in Zhang et al. [19] we also report Dawid-Sebastiani (DS) score which is defined in Equation (38)

(38) $$DSS_{t,i}\left(X_{t,i}\right)=\frac{X_{t,i}-\mu_{t,i}}{\sigma_{t,i}}+2\log\left(\sigma_{t,i}\right).$$

## 5. Simulation Examples

**Figure 3:**
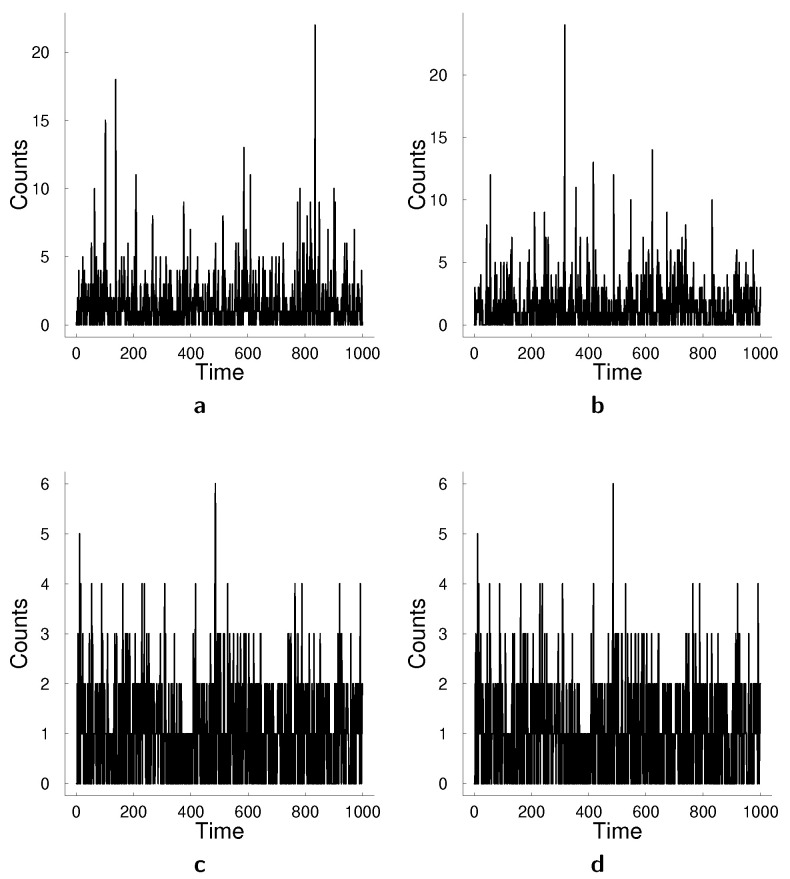
Examples of the data generated with the state-space and log-linear MACI models. (**a**) Dimension 1 of the bivariate time series generated from SSM2 in Table 2. (**b**) Dimension 2 of the bivariate time series generated from SSM2 in Table 2. (**c**) Dimension 1 of the bivariate time series generated from LL2 in Table 2. (**d**) Dimension 2 of the bivariate time series generated from LL2 in Table 2.

In this section, we demonstrate the performances of the models based on simulated data. We generate data from various specifications of SSM and log-linear MACI models and compare the models on forecasting performance. We assess forecasting performance based on six different scoring measures discussed in the previous section. The design of the simulation study allows us to assess forecasting performance in the cases of both correct model specification and misspecified case. Table 2 summarizes three different specifications of the state-space approach for data generation and Table 3 summarizes specifications of the log-linear MACI for data generation. Figure 3 illustrates two examples of bivariate time series generated from these models. For each simulation setting, we generate ten datasets with different random seeds and report the average results from these ten datasets. State-space model was estimated using particle Metropolis-Hastings algorithm with N=5000 particles and M=20000 Metropolis-Hastings step with a warm-up period of 5000 steps. The acceptance rate was targeted to be between 25% and 40%.

We assess the forecasting performance of two models for multivariate count data: state-space model and log-linear model. Table 4 and Table 5 summarize the forecasting performances of the models according to various scoring rules. The rows of the tables correspond to a particular data generating process (see for details of specification Table 2 and Table 3) and columns for a particular scoring rule (see scoring rules specification in Table 1). In particular, Table 4 shows performance of the state-space model and Table 5 the performance of the log-linear model. The state-space model outperforms the log-linear MACI model when the data are generated from $SSM_{1}$ (SSM with positive correlation) and $LL_{1}$ (log-linear model with a negative $a_{11}$ coefficient). It is particularly interesting that when the data is simulated from $LL_{1}$, SSM performs best according to all measures despite being a misspecified model. When the data are generated from $SSM_{2}$ and $SSM_{3}$, the state-space approach performs best based on most measures. This result is expected as SSM is the correct model specification for these simulated data. Finally, log-linear MACI model performs best in the case of data set $LL_{2}$—in the case when the model is correctly specified and all the coefficients are positive—according to most measures.

## 6. Empirical Applications

In this section, we compare the models in two empirical applications—bank failures and transactions data. These data sets exhibit different sample sizes, standard deviation, dispersion and maximum counts. In particular, bank failure time series reach a maximum of 10 and 24 counts while transaction data reach up to 67 and 60 counts with comparable mean counts.

### 6.1. Bank Failures

Bank failures have been analyzed using a univariate Poisson process [36]. A number of researches have investigated bank failure data of different time periods, see e.g., Schoenmaker [1] for an analysis of contagion risk in banking. Overall, it is reasonable to expect that bank failures in different countries are driven by similar economic phenomena, and possible contagion/spillover effects exist between economies of different countries.

**Figure 4:**
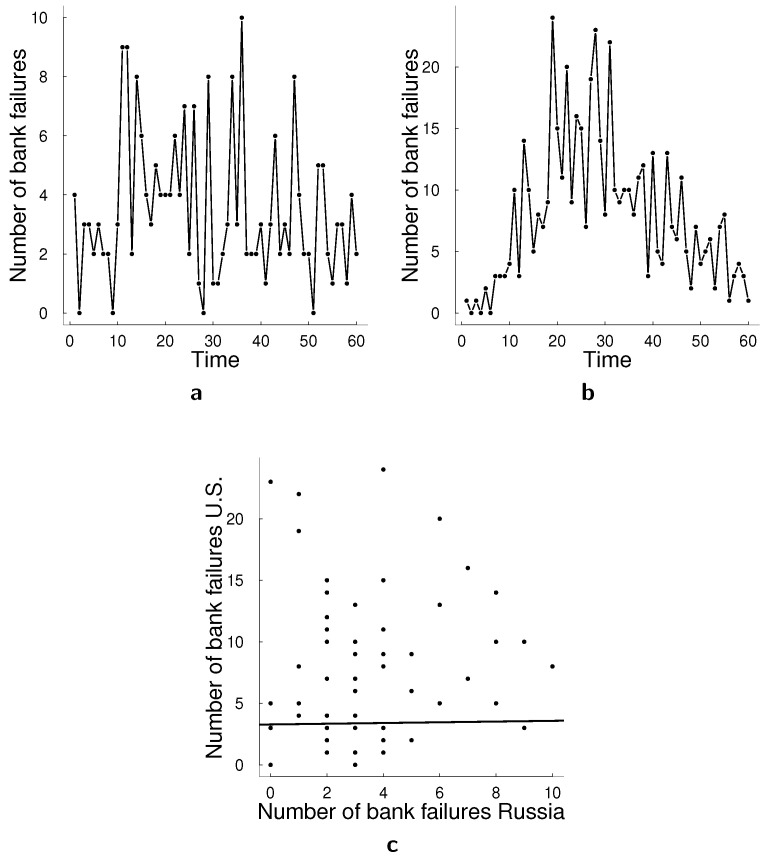
Data for bank failures empirical application. (**a**) Monthly bank failures in Russia January 2008–December 2012. (**b**) Monthly bank failures in the U.S. January 2008–December 2012. (**c**) Scatter plot of data bank failures in subplots (**a**,**b**).

For this application, we analyze count data using a bivariate data set of bank failures in the U.S. and Russia that has not been considered in the literature before. We use monthly number of bank failures for the period between January 2008 and December 2012 for both countries and apply the bi-variate specifications of the models in Section 2 and Section 3. Especially due to the global financial crisis included in this period, it is important to allow for potential correlation between the number of bank failures in the U.S. and Russia using the multivariate count data models. Figure 4 illustrates these time series and Table 6 presents descriptive statistics for this data set.

The estimation results from both models are presented in Table 7 and Table 8. Based on the state-space model, the correlation is estimated as being low negative and 0 is included in highest posterior density interval for this parameter. Despite that log-linear MACI model estimates correlation coefficient to be positive, it provides a large confidence interval for this parameter which also includes 0. Thus, for this relatively small data set, we do not find an indication of correlated bank failures using both models. We also note that some confidence intervals in Table 8 include point 0. As discussed in Section 2, applying observation driven models with positivity constraints would be problematic for these data especially in terms of the calculation of confidence intervals.

We next compare the models in terms of their forecast performances. For this comparison, we take a sample size of T=55, and we make five steps ahead predictions using the log-linear model and the state-space approach. Table 9 presents scores for this forecasting exercise. Based on all scores, except for the rank probability score (rps), the state-space model outperforms the log-linear model in terms of forecasting. Based on the simulation results in Section 5, we conjecture the following: The better performance of the state-space model can be due to this model being close to the true data generating process, or due to its property of capturing data properties well even if it is misspecified.

### 6.2. Transactions

**Figure 5:**
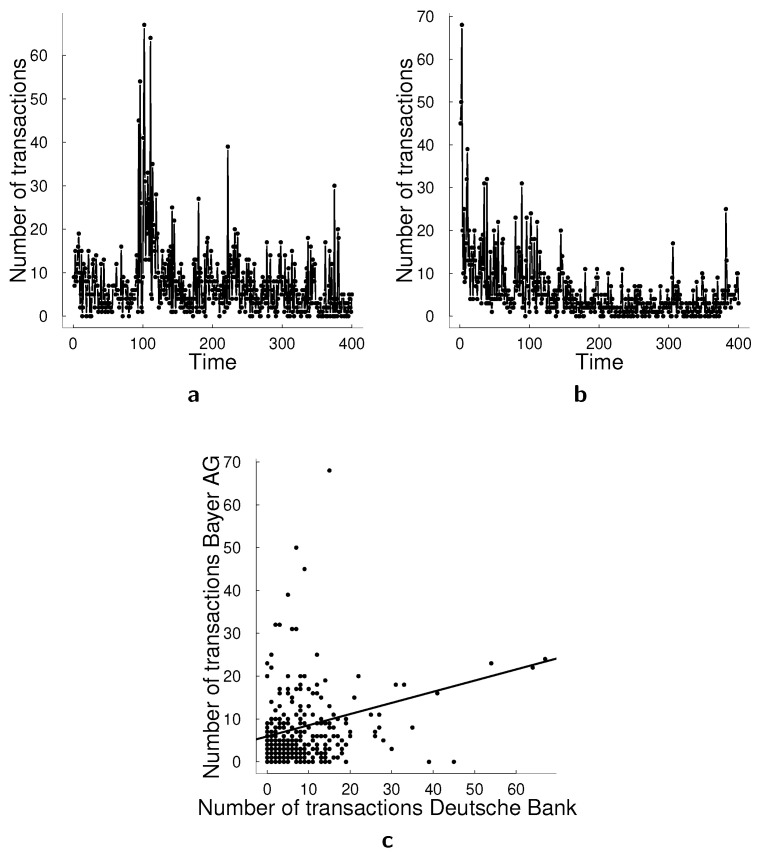
Data for transactions empirical application. (**a**) Transactions in 30 s. interval for Deutsche Bank AG. (**b**) Transactions in 30 sec. interval for Bayer AG. (**c**) Scatter plot of transactions in (**a**,**b**).

In this empirical application, we analyze the number of transactions on 30 s intervals for Deutsche Bank AG and Bayer AG (the datawere obtained from FactSet, in the time period of 3 August 2015 09:05:30 until 3 August 2015 12:25:00 for the training data). We expect such transactions to be correlated due to their dependence on the time of the day and the market conditions. The sample size in this application is T=400, which is significantly larger than the sample size in the bank failures application. The summary statistics for this data set are provided in Table 10 and Figure 5 illustrates these time series. Both time series have fat tails with a few very high values, concentrated around observation 100 and 1 for Deutsche Bank and Bayer AG, respectively.

We apply the bi-variate counterparts of the count data models in Section 2 and Section 3 to these data and compare the model performances based on 100 steps ahead forecasts. In Table 11 and Table 12 we present parameter estimates of both models. Both models estimate positive correlation between these time series. However, in the case of the log-linear MACI model the estimated correlation coefficient is much higher. In addition, the confidence intervals of parameter estimates such as $b_{12}$ and $b_{22}$ in Table 12 include point 0. Thus, true parameters being non-positive is a potential problem if other observation driven models, with positivity constraints, in Section 2 were applied to these data.

**Figure 6:**
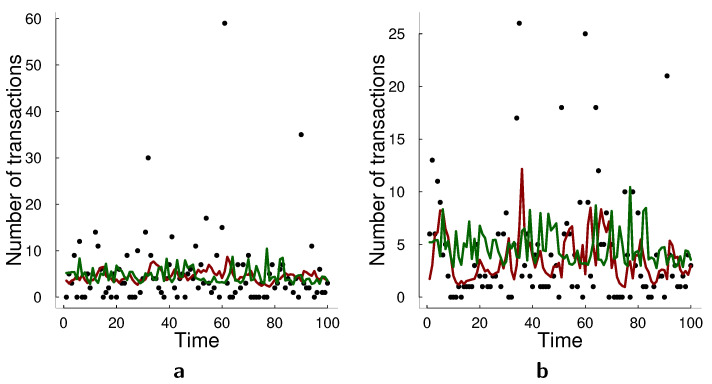
Forecasts and true data for the transactions empirical application. (**a**) Transactions in 30 s. interval for Deutsche Bank AG (black circles), forecast with SSM (green), forecast with log-linear MACI (red). (**b**) Transactions in 30 s. interval for Bayer AG (black circles), forecast with SSM (green), forecast with log-linear MACI (red).

Table 13 presents the scores of each model in the forecast sample. In this application, the log-linear model performs best according to all scoring rules. Based on the simulation results in Section 5, we conjecture that the log-linear model is potentially closer to the true data generating process compared to the state space model. We further analyze the forecasting performances of the models in Figure 6. Particularly in Figure 6b, we observe that the log-linear MACI model captures better high spikes of the counts and then returns to the original level of the data. The forecast with the state-space model appears to be too smooth compared to the data points. Thus, the better forecast performance of the log-linear MACI is potentially due to its ability to capture these extreme data values successfully.

The under-performance and over-smoothing of the state-space approach can be mitigated by implementing a different particle filter. For example, one could take the direction of implementing look-ahead particle filters such as [37,38]. General idea of the look-ahead approaches is that in the particle filtering algorithm we make a proposal not just according to the dynamics of the model $p(h_{t}|h_{t-1})$, but taking the current observation into account $p(h_{t}|h_{t-1},y_{t})$ or taking into account the complete time series $p(h_{t}|h_{t-1},y_{1:T})$ as in [38]. These methods, however, have not been developed for the distributional assumptions we are considering in this paper and further research is needed in this direction.

## 7. Discussion

In this paper, we have reviewed and compared two approaches for modeling multivariate count time series data. One of the challenges that appears in the literature and have not been resolved is modeling the dependency between counts in a flexible way that would also allow for feasible estimation. We have discussed multivariate autoregressive conditional intensity models (MACI), their log-linear alternative which we refer to as the multivariate log-linear model and nonlinear state-space model. Both models have advantages and disadvantages. In particular, the nonlinear state-space framework allows for various interpretable dependencies that one cannot easily incorporate into MACI or log-linear approach. However, these models can be computationally expensive to estimate, in particular in higher-dimensions. Challenges in estimation arise from different sources. State-space models naturally fit into the Bayesian framework, however, since both the likelihood and the posterior of the model are analytically intractable this leads to computationally expensive procedure. MACI models, on the other hand, are quite restrictive: they restrict coefficients in the model to be positive as well as the correlation between time series. Both assumptions can be unrealistic in many real-world applications. Log-linear model avoids the problem of only positive coefficients in the model by logarithmic transformation of the data. However, estimation can be unstable, and good starting points need to be chosen for the estimation. When the dimension of the model grows, it becomes harder to choose good starting points for the optimization problem. The computational advantage of log-linear and MACI models decreases with the increase in either dimensionality of the model or the number of counts. This reduction in the computational advantage is due to the usage of the multivariate Poisson distribution as every pairwise correlation has to be modeled as a separate Poisson random variable. Moreover, the summation in the specification of the joint distribution runs through the number of counts. Generally, one could say that estimation of log-linear models much faster than of the state-space models. In low dimensions and with the small number of counts these models do not require much of computational power, however, once the number of counts increases and once we deal with higher dimensions, the computations become much more extensive due to large sums in the multivariate Poisson distribution. Moreover, while running the model on simulated and empirical data, we found that the estimation can be numerically unstable and can highly depend on the starting values in the estimation procedure. We follow the suggestion of Doukhan et al. [10], and the first estimate the model for univariate time series. These estimates we further use in multivariate estimation. However, the problem of numerical instability especially remains in small samples according to our experience. Nevertheless, in terms of flexibility, this model is the best competitor for the state-space approach.

We have compared log-linear models and state-space models for count data in terms of forecasting performance on multiple simulated data sets and real data applications. We found that on the simulated data state-space framework generally outperforms log-linear model, sometimes even under model misspecification. On the real data sets, the state-space model performs better in bank failures applications which consists of two time series of bank failures in Russia and U.S. and the counts remain relatively low and the data are relative smooth. The log-linear model performs better in the transactions applications in which we consider two time series of transactions counts in 30 s intervals. The challenge of transactions application is that there are spikes of counts which deviate a lot from the mean. In this case, we notice that the log-linear model approximates these spikes better. Thus, a possible direction for future research is adapting a multivariate state-space model for count data to capture such spikes better. A possible way to improve the model in this regard would be to adapt the particle filtering algorithm. We used bootstrap particle filter which does not take into account observations when making a proposal for particles, but taking current (or all) observation into account in the proposal mechanism for the particles can help approximating the spikes in the data. There have been proposed multiple look-ahead approaches for particle filters [37,38], but they have not been adapted to count data.

Finally, both approaches have their drawbacks. In particular, the log-linear model seems to have numerical stability issues and finding optimal starting values for optimization can be a challenge. In the state-space approach, the challenging part is the estimation of the likelihood, which is intractable and sampled from the posterior distribution. Additionally, the state-space model in its current implementation is challenged by possible spikes in the data to a larger degree than the log-linear model.

## Figures and Tables

**Table 1:** Scoring rules for assessment of the forecasts. The table summarizes scoring rules that we use to assess forecasting performance of the models under consideration, proposed in Czado et al. [35] for count data.

Logarithmic score	$\log\left(P,x\right)=-\log p_{x}$
Quadratic score	$qs\left(P,x\right)=-2p_{x}+||p|{|}^{2}$
Spherical score	$sphs\left(P,x\right)=-\frac{p_{i}}{||p||}$,
	where $||p|{|}^{2}=\sum_{k=0}^{\infty }p_{k}^{2}$
Rank probability score	$rps\left(P,x\right)=\sum_{k=0}^{\infty }{\left\{P_{k}-1\left(x\geq k\right)\right\}}^{2}$
Squared error score	$ses\left(P,x\right)={\left(x-\mu_{p}\right)}^{2}$,
	where $\mu_{p}$ is the mean of *P*

**Table 2:** True parameters for the data sets generated from the state-space model in the simulation examples. All data generating processes include a one-directional Granger-causal feedback through a non-zero coefficient $\phi_{21}$ and different correlation structures: $SSM_{1}$ has a positive correlation coefficient ρ, $SSM_{2}$ has a negative correlation coefficient ρ and $SSM_{3}$ has no correlation.

Data Set	$\beta_{1}$	$\beta_{2}$	$\phi_{11}$	$\phi_{21}$	$\phi_{12}$	$\phi_{22}$	$\sigma_{\eta_{1}}$	$\sigma_{\eta_{2}}$	ρ
$SSM_{1}$	1.0	2.0	0.5	0.3	0.0	0.5	0.5	0.5	0.3
$SSM_{2}$	1.0	2.0	0.5	0.3	0.0	0.5	0.5	0.5	−0.3
$SSM_{3}$	1.0	2.0	0.5	0.3	0.0	0.5	0.5	0.5	0.0

**Table 3:** True parameters for the data sets generated from the log-linear MACI model in the simulated examples.

Data Set	$\omega_{1}$	$\omega_{2}$	$a_{11}$	$a_{22}$	$b_{11}$	$b_{12}$	$b_{21}$	$b_{22}$
$LL_{1}$	0.9	0.4	−0.5	0.2	0.5	0.2	0.0	0.4
$LL_{2}$	0.2	0.3	0.2	0.4	0.5	0.2	0.0	0.4

**Table 4:** Scores for the forecasting exercise with the state-space model, according to the definitions in Table 1. The smaller score indicates a better result. DGP column corresponds to the data generating processes in this simulation study, the true parameters are presented in Table 2 and Table 3.

DGP	log	qs	sph	rps	ds	se
$SSM_{1}$	**1.484**	**−0.229**	**−0.440**	**0.770**	**2.352**	**2.634**
$SSM_{2}$	**1.861**	**−0.235**	**−0.487**	**1.000**	3.136	**3.551**
$SSM_{3}$	1.967	−**0.224**	**−0.475**	**0.948**	3.599	**4.075**
$LL_{1}$	**1.959**	**−0.164**	**−0.405**	**0.974**	**2.176**	**3.214**
$LL_{2}$	1.351	−0.293	−0.543	**0.545**	1.103	1.087

**Table 5:** Scores for the forecasting exercise with the log-linear MACI model. The smaller score indicates a better result. DGP column corresponds to the data generating processes in this simulation study, the true parameters are presented in Table 2 and  Table 3.

DGP	log	qs	sph	rps	ds	se
$SSM_{1}$	1.636	−0.321	−0.553	0.999	2.612	3.088
$SSM_{2}$	2.089	−0.164	−0.391	1.333	**2.614**	5.180
$SSM_{3}$	**1.929**	−0.220	−0.469	**0.948**	**3.464**	4.187
$LL_{1}$	1.985	−0.159	−0.400	0.996	2.238	3.357
$LL_{2}$	**1.320**	**−0.309**	**−0.555**	0.555	**1.036**	**1.023**

**Table 6:** Descriptive statistics for the bank failures data for the period January 2008 until December 2012 for Russia and U.S.

	Russia	U.S.
mean	3.51	7.93
median	3	7
st.d.	2.46	5.93
minimum	0	0
maximum	10	24

**Table 7:** Posterior moments of the parameters of the state-space model for bank failures.

	Mean	Median	Mode	${\mathrm{HPD}}_{l}$ 95%	${\mathrm{HPD}}_{u}$ 95%
$\beta_{1}$	1.1450	0.8867	0.0503	0.0503	2.9481
$\beta_{2}$	4.0414	3.7053	2.3414	1.8570	7.2843
$\phi_{11}$	0.9569	0.9648	0.9757	0.8968	0.9991
$\phi_{21}$	0.0101	0.0071	−0.0372	−0.0646	0.0856
$\phi_{12}$	0.1193	0.0935	−0.1856	−0.2367	0.5438
$\phi_{22}$	0.7387	0.7518	0.8862	0.4942	0.9733
$\sigma_{\eta_{1}}$	0.3335	0.3340	0.0122	0.1722	0.5400
$\sigma_{\eta_{2}}$	0.3302	0.3252	0.3189	0.1496	0.5176
ρ	−0.0845	−0.0848	−0.0703	−0.1879	0.0209

**Table 8:** Parameter estimates of the log-linear MACI model for bank failures.

	Estimate	${\mathrm{CI}}_{l}$ 95%	${\mathrm{CI}}_{u}$ 95%
$w_{1}$	0.1259	−0.7545	1.0064
$w_{2}$	−0.1307	−0.4348	0.1733
$a_{11}$	0.0732	−0.5380	0.6844
$a_{22}$	0.6923	0.5535	0.8312
$b_{11}$	0.0403	−0.2816	0.3621
$b_{21}$	0.1638	0.0190	0.3086
$b_{12}$	0.3879	0.0212	0.7546
$b_{22}$	0.2521	0.1069	0.3974
ρ	0.6513	−0.2131	1.5158

**Table 9:** Scores for the forecasting exercise with bank failure data. This table shows scoring rules for the forecasting exercise in the bivariate model for bank failure data.

Model	log	qs	sph	rps	ds	se
SSM	**1.9026**	**−0.1738**	**−0.4189**	0.9755	**2.1619**	**3.0841**
Log-Linear	2.0244	−0.1623	−0.3996	**0.8862**	2.2934	4.2031

**Table 10:** Descriptive statistics for the transactions data.

	Deutsche Bank AG	Bayer AG
mean	6.95	7.716
median	5	5
st.d.	8.2462	8.227
minimum	0	0
maximum	67	60

**Table 11:** Posterior moments of the para maters for the state space model for transactions.

	Mean	Median	Mode	${\mathrm{HPD}}_{l}$ 95%	${\mathrm{HPD}}_{u}$ 95%
$\beta_{1}$	4.5049	4.4860	4.3782	3.9274	5.1238
$\beta_{2}$	5.4475	5.4260	5.2161	4.7971	6.1541
$\phi_{11}$	0.3058	0.3048	0.3137	0.1778	0.4316
$\phi_{12}$	0.0180	0.0181	−0.0469	−0.1007	0.1342
$\phi_{21}$	0.0518	0.0533	0.1520	−0.1118	0.1890
$\phi_{22}$	0.3788	0.3795	0.5341	0.2414	0.5126
$\sigma_{\eta_{1}}$	0.8864	0.8853	0.8759	0.8059	0.9675
$\sigma_{\eta_{2}}$	0.7521	0.7519	0.7246	0.6835	0.8236
ρ	0.2400	0.2397	0.2499	0.1932	0.2875

**Table 12:** Parameter estimates of the log-linear MACI model for transactions.

	Estimate	${\mathrm{CI}}_{l}$ 95%	${\mathrm{CI}}_{h}$ 95%
$w_{1}$	0.1232	0.0304	0.2161
$w_{2}$	−0.0741	−0.1666	0.0184
$a_{11}$	0.7518	0.6826	0.8211
$a_{22}$	0.5333	0.4586	0.6080
$b_{11}$	0.1832	0.1471	0.2193
$b_{21}$	0.0315	−0.0028	0.0659
$b_{12}$	0.0024	−0.0256	0.0305
$b_{22}$	0.4591	0.3847	0.5334
ρ	0.7967	0.6053	0.9881

**Table 13:** Scores for transaction forecasts.

Model	log	qs	sph	rps	ds	se
SSM	5.0	−0.0171	−0.2059	3.4601	14.5471	49.97
Log-Linear	**4.4549**	**−0.0232**	**−0.2152**	**3.1621**	**11.3674**	**44.429**

## Data Availability

The data for Polio and Asthma cases is publicly available from *R* library glarma [39]. Number of car crashes previously published in [40]. The earthquakes data is available from https://earthquake.usgs.gov/ (accessed on 14 June 2017). Transactions data can be downloaded from FactSet. The data for bank failures is publicly available at www.banki.ru (Russia) (accessed on 2 June 2021) and www.fdic.gov (U.S.) (accessed on 2 June 2021).

## Abbreviations

The following abbreviations are used in this manuscript:

SSM	State-Space Model
MACI	Multivariate Autoregressive Conditional Intensity
INGARCH	Integer-Valued Generalized Autoregressive Conditional Heteroskedastic Model
MCMC	Markov Chain Monte Carlo
PMCMC	Particle Markov Chain Monte Carlo
SMC	Sequential Monte Carlo
